# Biomechanical evaluation of novel intra- and extramedullary assembly fixation for proximal humerus fractures in the elderly

**DOI:** 10.3389/fbioe.2023.1182422

**Published:** 2023-10-23

**Authors:** Zhengguo Zhu, Zuhao Chang, Wei Zhang, Hongzhe Qi, Hao Guo, JiaQi Li, Lin Qi, Shaobo Nie, Peifu Tang, Yonghui Liang, Xing Wei, Hua Chen

**Affiliations:** ^1^ The Department of Orthopaedic, Aerospace Center Hospital, Beijing, China; ^2^ The Department of Orthopaedic Trauma, Chinese PLA General Hospital (301 Hospital), Beijing, China; ^3^ The Department of Orthopaedic Surgery, Beijing Chaoyang Hospital, Capital Medical University, Beijing, China; ^4^ AI Sports Engineering Lab, School of Sports Engineering, Beijing Sport University, Beijing, China; ^5^ The Department of Orthopaedic Trauma, Strategic Support Force Medical Center, Beijing, China; ^6^ The Second Surgical Department, Beijing Municipal Corps Hospital of Chinese People’s Armed Police Force, Beijing, China

**Keywords:** biomechanical analysis, proximal humeral fractures, osteopenia, locking plate fixation, intramedullary nail, hybrid fixation

## Abstract

**Purpose:** A novel intra- and extramedullary assembly fixation method was introduced, which achieved good clinical results in complex proximal humeral fractures; however, evidence of its comparability with traditional fixation is lacking. This biomechanical study aimed to compare it with traditional fixation devices in osteoporotic proximal humeral fractures.

**Methods:** Three-part proximal humeral fractures with osteopenia were created on 12 pairs of fresh frozen humerus specimens and allocated to three groups: 1) lateral locking plate, 2) intramedullary nail, and 3) intra- and extramedullary assembly fixation. The specimens were loaded to simulate the force at 25° abduction. Thereafter, an axial stiffness test and a compound cyclic load to failure test were applied. Structural stiffness, number of cycles loaded to failure, and relative displacement values at predetermined measurement points were recorded using a testing machine and a synchronized 3D video tracking system.

**Results:** In terms of initial stiffness and the number of cycles loaded to failure, the intra- and extramedullary assembly fixation group showed notable improvements compared to the other groups (*p* <0.017). The mean relative displacement value of measurement points in the intra- and extramedullary assembly fixation group was smaller than that in the other two groups. However, there was no significant difference until 10,000 cycles. The mean relative displacement of the intramedullary nail group (3.136 mm) exceeded 3 mm at 7,500 cycles of loading.

**Conclusion:** In this test model, axial fixation can provide better mechanical stability than non-axial fixation. The intra- and extramedullary assembly fixation is better able to prevent the varus collapse for elderly proximal humeral fractures with posteromedial comminution.

## Introduction

Proximal humeral fractures (PHFs) are the third most common fragility fractures in the elderly with regard to the increasing cases of osteoporosis (Karl et al., 2015). Due to thinning trabeculae and cortical bones, severely displaced and comminuted PHFs with medial calcar impaction are common injuries and are normally treated using a locking plate or intramedullary nail internal fixation (Mease et al., 2021). Unfortunately, complications, such as a loss of reduction and internal fixation failure, and functional limitations occurred in approximately 40% of patients who underwent surgery, and the reoperation rate was 19.7% (Barlow et al., 2020; Bergdahl et al., 2021). Increasing the mechanical stability of the medial column and counteracting varus displacement force may be useful to avoid those complications (Euler et al., 2017; Dasari et al., 2022).

Therefore, both lateral locking plates (LLPs) and intramedullary nails (IMNs) add inferomedial screws to optimize the support position to extend medially (Erdoğan et al., 2014; Katthagen et al., 2015; Wanzl, 2016). Even so, the inherent non-axial fixation structure of the LLP makes it challenging to provide sufficient structural stability for osteoporotic PHFs with an unstable medial column, resulting in complications, such as screw penetration of the joint and humeral head varus deformity (Lorenz et al., 2021). As an axial fixation method, IMNs show better mechanical stability in transmitting the vertical force of the medial humerus and maintaining the neck-shaft angle (Füchtmeier et al., 2007; Kitson et al., 2007; Gradl et al., 2009). Moreover, the entry point is moved medially to increase stability by anchoring it to the densest zone of the proximal humerus (subchondral zone) (Euler et al., 2017). However, due to the method of implantation and osteoporosis, the complication rate of a rotator cuff tear, the poor reduction of the greater tubercle, and the retraction of the nail from the entry point reached 33.3%, which seriously affects the shoulder joint activity of patients (Gradl et al., 2009; Plath et al., 2019). The optimal surgical strategy for elderly proximal humerus fractures is still an unsolved problem in orthopedic surgery.

To solve the problem, a novel combined intramedullary support nail and an extramedullary plate fixation device were introduced; the intramedullary nail was shaped according to the medullary cavity’s geometry. The sliding head screw and distributed locking screw design prevent the screw from penetrating the joint (Bai et al., 2022). To the best of our knowledge, this is the first attempt at combining the advantages of the locking plate, intramedullary support, and dynamic screw fixation, and there are no biomechanical data. Therefore, the purpose of this study was to investigate and compare the biomechanical properties of three different types of fixations: 1) lateral locking plate, 2) intramedullary nail, and 3) intra- and extramedullary assembly fixation (IEAF). It was hypothesized that the IEAF can improve the biomechanical stability in the treatment of elderly PHFs with posteromedial comminution.

## Materials and methods

### Specimens

Twelve pairs of fresh frozen humeri of 12 donors (six male and six female subjects; mean age: 70.3 years and range: 60–83) were obtained from the local anatomical department. A peripheral quantitative computed tomography scan (pQCT-scan) (GE LightSpeed VCT 16, Milwaukee, United States) was performed to rule out any prior surgery and pre-existing pathologies, with the equal ratio of male-to-female subjects and left-to-right sides in each group. Specimen demographics, the diameter of the humeral head, and bone mineral density (BMD) are shown in Table 1. The bones were frozen at −20°C until further processing.

**TABLE 1:** Specimen demographics and BMD and the diameter of the humeral head.

	Extramedullary group	Intramedullary group	Hybrid group	*p*-value
Gender (M/F)	1	1	1	1
Age (year)	65.25 ± 7.304	66.375 ± 9.44	64.375 ± 7.405	0.886
Bone mineral density (BMD mgHA/mL)	57.413 ± 7.44	60.013 ± 8.532	54.425 ± 9.113	0.426
Diameter of the humeral head (mm)	45.175 ± 0.824	45.075 ± 1.075	45.639 ± 0.792	0.447

### The biomechanical model

#### Specimen preparation

Before instrumentation, the specimens were thawed overnight at 4°C in a refrigerator.

**FIGURE 1:**
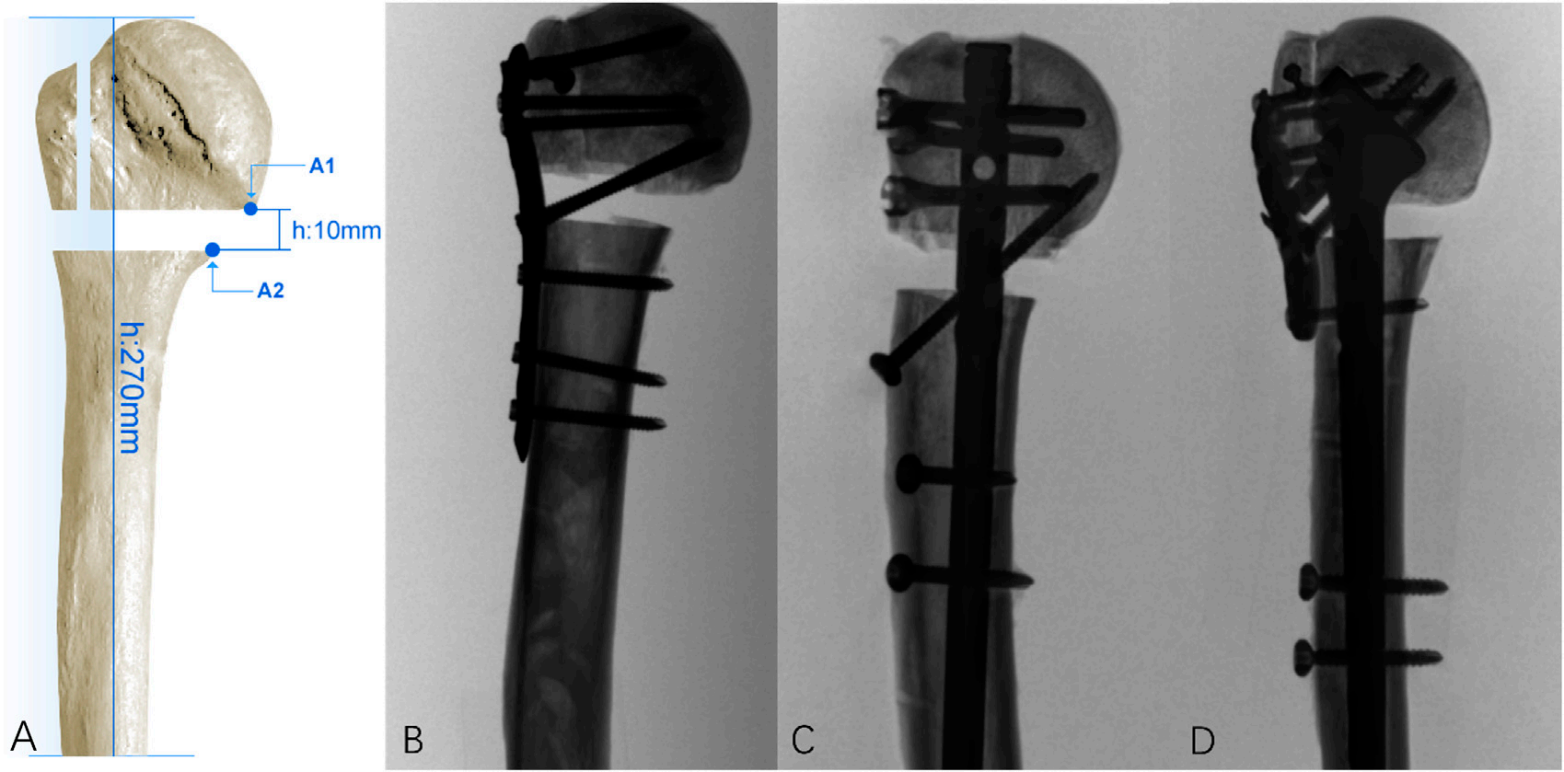
**(A)** 10-mm horizontal segmental bone defect below the surgical neck. The distance between the distal end of the humerus and the apex of the humeral head is 270 mm, and a pair of points on the opposite side of the fracture site is located on the most medial position of the surgical neck ostectomy (A1–A2); **(B)** in the LCP group, the plate was secured to the lateral proximal humerus with three distal locking screws and four proximal screws with two calcar screws; **(C)** in the IMN group, the nail was secured to the proximal humerus with two distal screws and three proximal screws and one calcar screw; **(D)** in the IEAF group, the intramedullary strut was secured to the humerus with two distal screws and three sliding screws, and the plate was secured to the lateral proximal humerus with one distal locking screw and four proximal screws.

All the samples were prepared in five steps: 1) dissection of the surrounding soft tissue; 2) standardization of the shaft length: all the samples were reduced to an equal length, cutting the shaft perpendicular to its axis 270 mm distal to the apex proximal head; 3) a three-part PHF was simulated with a greater tuberosity osteotomy and surgical neck osteotomy using an oscillating saw blade with a thickness of 0.4 mm; 4) making a 10-mm horizontal segmental bone defect below the surgical neck (Figure 1A); 5) a custom-made anchor was made on the lateral-posterior side of the greater tuberosity fragment. Two sutures (Ethicon W4843) were connected with surgical knots to that custom-made anchor. During preparation, each specimen was covered with a towel soaked in saline solution and was periodically sprayed with saline solution to prevent desiccation.

#### Instrumentation

**FIGURE 2:**
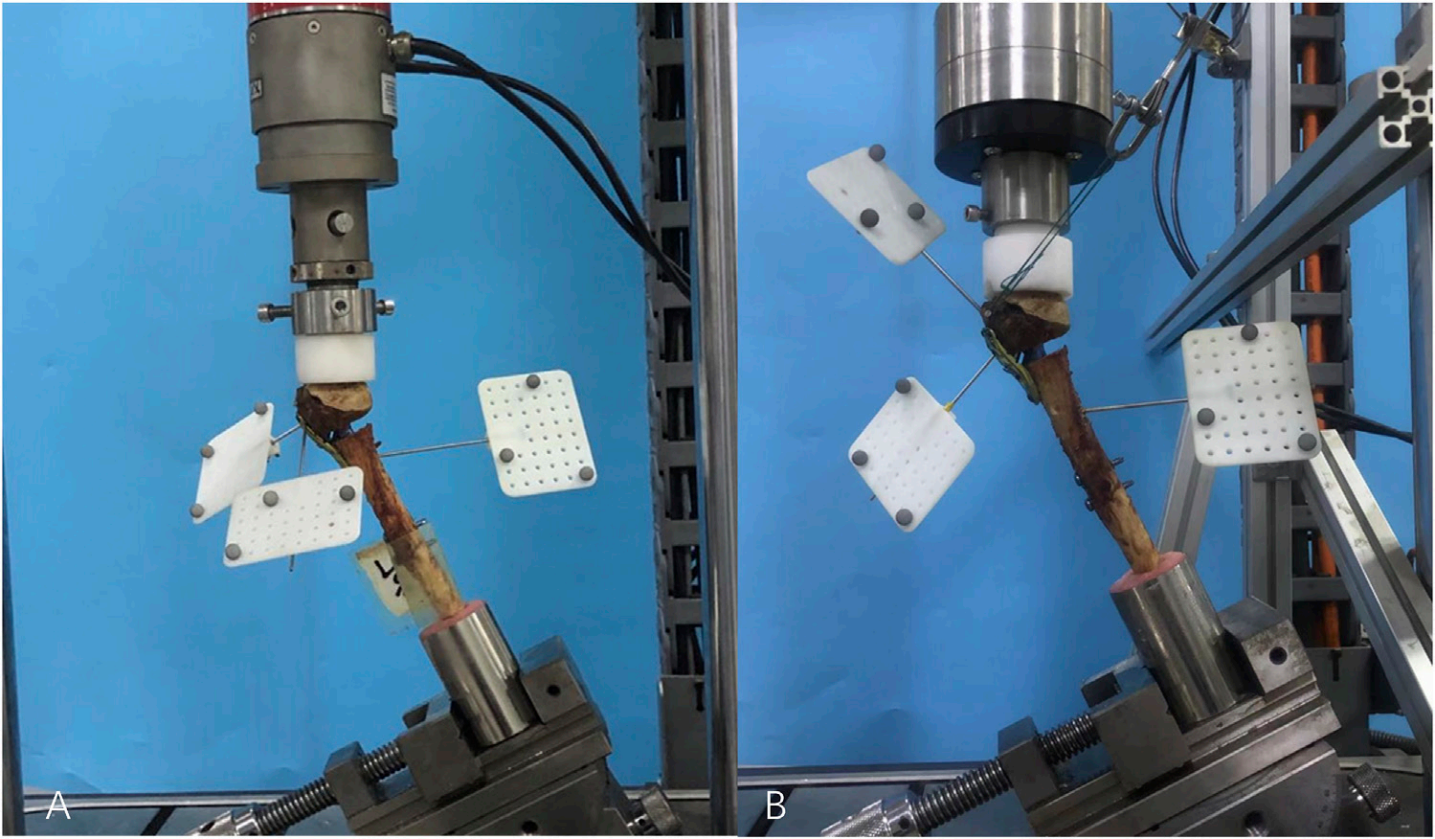
**(A)** Axial stiffness test**,** an adjustable angle vise constrained each sample at 25° angles to the machine; **(B)** in the compound cyclic load until failure test, three markers were located on the humeral shaft, humeral head, and greater tuberosity. The pulling force acting on the anchor inserted in the greater tuberosity fragment was angled at 110° to the axis of the humeral shaft in the medial-lateral plane.

**FIGURE 3:**
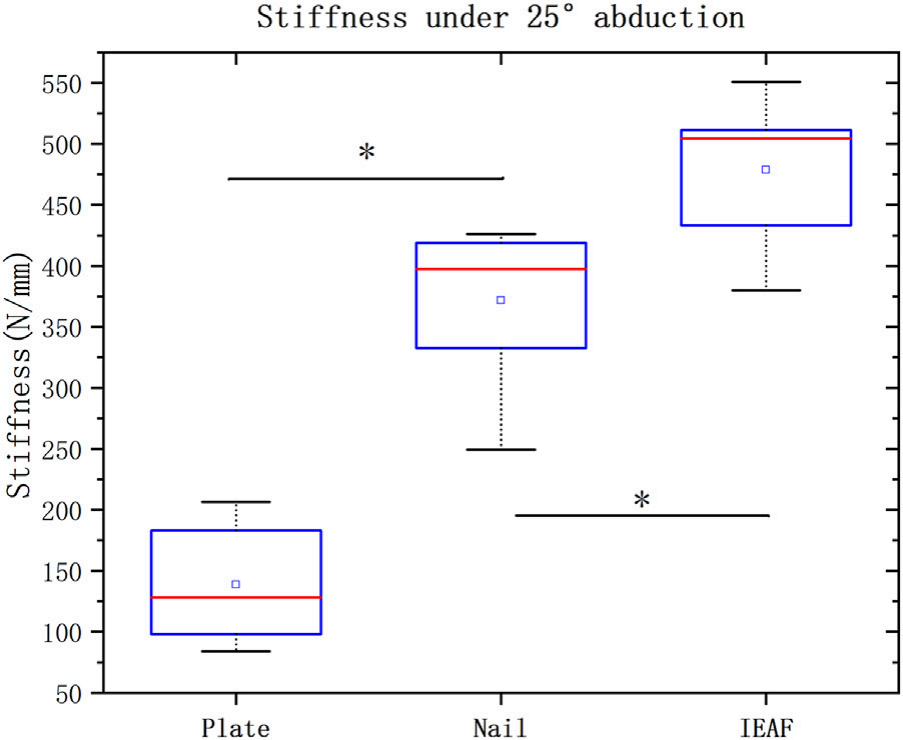
Box and whisker plots showing the values of axial stiffness in N/mm. The horizontal red line in each box indicates the median value; the top and bottom borders of the box show the 75th and 25th percentiles, respectively; the whiskers show the 10th and 90th percentiles. Significant differences are indicated as *. The square symbols represent the mean.

Three fixation constructs were tested, and fluoroscopy (First-Imaging Ltd., Jiangsu) was used to verify the proper implant placement and fracture fixation (Figure 1): 1) the plate group: a locking plate (PHILOS, Synthes GmbH, Switzerland) (Figure 1B); 2) the nail group: intramedullary nails (MultiLoc, Synthes GmbH, Switzerland) (Figure 1C); 3) the IEAF group: a novel intra- and extramedullary assembly fixation device (Figure 1D).

Standard surgical techniques instrumented the fixation constructs into the samples. For PHILOS, the plate was secured to the lateral proximal humerus with three distal locking screws, four proximal screws, and two calcar screws; for MultiLoc, the nail was secured to the humerus with two anti-rotational locking screws, three proximal screws, and one calcar screw. For the IEAF system, the intramedullary strut was instrumented into the endosteal of the proximal humerus and was secured to the bone with two distal screws. The extramedullary plate was secured to the humerus with four distributed locking screws, which were far from the articular surface’s center, and one distal locking screw. Three sliding screws pass through both the extramedullary plate and the intramedullary strut that prevents the screws from penetrating the joint. All the procedures, including drawing, osteotomy, and implant fixation, were performed by the same surgeon.

### Biomechanical setup

A testing machine (Model E10000; Instron, Norwood, Massachusetts) was used to perform mechanical loading. We adopted a loading methodology similar to those mentioned by Stefano et al. (Brianza et al., 2010; Zhu et al., 2023). The distal humerus was encapsulated by polymethyl methacrylate (PMMA) cement in an 8-cm-long stainless-steel tube. A distally adjustable angle vise connected to the base-plate restrained all the sample displacements and the rotation around the shaft axis. A three-dimensional video tracking system (Noitom Ltd., Beijing) was used to track the three-dimensional motion of the selected points on the fracture site and to measure the displacement of the spatial coordinates. A pair of points on the opposite side of the fracture site located at the most medial position of the surgical neck ostectomy (A1–A2) (Figure 1A) was considered of interest, and their relative displacement was calculated in the three-dimensional spatial coordinates. The spatial resolution of the system is in the order of 0.15 mm.

#### Axial stiffness test

Each sample was restrained to the machine with 25° lateral angulations (Figure 2A) (Bergmann et al., 2007). Before each test, 10 settling cycles of 0–50 N at a machine velocity of 0.02 mm/min were applied to the specimen, which aimed to achieve a relatively stable state between the system and the constructs. Axial loading from 50 to 200 N in displacement control (0.02 mm/s) was applied to the samples using a spherically shaped PMMA shell cup (the inner diameter of the cup is large enough to restrict the movement of the humeral head) attached to the load cell. The stiffness was determined by calculating the average slope of the linear portions of force–displacement curves.

#### Compound cyclic load until failure

The PMMA cup was attached to the machine’s actuator via a custom flange that transmits only the axial load to the sample (Figure 2B). The rotational bearing associated with the custom-made flange allowed the decoupling of the effect of actuator axial and torsional movements. The torsional actuator was then used to independently generate the force pulling on the greater tuberosity fragment via a cable. The initial direction of the pulling force acting on the anchor inserted in the greater tuberosity fragment was angled at 110° to the axis of the humeral shaft in the medial-lateral plane (Figure 2B). An increasing axial compression load was applied at 2 Hz, in phase with pulling on the bone anchor. Starting from 200 N, the maximum (peak) of the curve was cyclically increased (0.05 N/cycle) until failure, while its minimum (valley) was kept constant at 50 N during the entire test. The pulling force (ranging from 40 to 100 N), acting on the anchor, simulated the destabilizing effect of a part of a rotator cuff tendon on the greater tuberosity [according to Favre (Favre et al., 2005)]. The test was automatically stopped when the fracture gap was closed or the fixation clearly failed.

### Data collection

For the axial stiffness test, we defined the axial stiffness (in N/mm) of the construct as the slope of force–displacement curves generated from the test system. For the compound cyclic load until failure test, the relative displacement (in millimeters) of the pair of points crossing the fracture lines was recorded when the number of cycles was 5,000 (the maximum load was 450 N) and 10,000 (the maximum load was 700 N), and the number of cycles when it fails was also recorded.

### Statistical analysis

The values for axial construct stiffness, displacement, and the number of cycles on failure were used for statistical analysis, which was performed using SPSS (IBM SPSS Statistics 22.0, SPSS Inc., Chicago, IL). The significance level was set to 0.05. After the testing of the normal distribution with the Shapiro–Wilk test, the study groups were then compared using Levene’s test and one-way ANOVA. If multiple comparisons showed a significant difference, Tamhane’s T2 *post hoc* analysis was carried out, following the one-way ANOVA. The Kruskal–Wallis test was used for data on non-normal distributions. The Bonferroni correction was utilized in these *post hoc* pairwise comparisons, with *p* <0.017 being considered significant. According to the general requirements of the statistics, taking *α* = 0.05 and *β* = 0.1, the pre-experiment illustrates that the mean ± standard deviation of the axial stiffness (the main index) in three different fixation groups was 138.68 ± 43.48 N/mm, 371.57 ± 61.45 N/mm, and 478.63 ± 57.96 N/mm. The aforementioned parameters were substituted into PASS 15 software, and it was concluded that the minimum number of cases to be completed in each group is three cases.

## Results

### Axial stiffness test

The initial axial stiffness of all three fixation constructs under the 50–200 N loading at 25° abduction is presented as the median and the range and as the mean standard deviation in Table 2, respectively. Box and whisker plots are depicted with *p*-values when the differences were significant in Figure 3. The IEAF demonstrated the greatest stiffness (478.625 ± 57.961 N/mm), a significant difference from the locking plate (138.6816 ± 43.484 N/mm; *p* <0.001), and the intramedullary nail (371.575 ± 61.445 N/mm; *p* = 0.014). There was a significant difference between the locking plate and intramedullary nail (*p* < 0.001).

**TABLE 2:** Axial stiffness for all three constructs.

	Plate	Nail	Hybrid
Mean	138.6816 ± 43.484	371.575 ± 61.445	478.625 ± 57.961
Median	128.485 (84.083–206.4)	397.41 (249.24–425.99)	504.59 (379.92–550.73)
*p*-value for the multiple-group comparison		*p* <0.001	
*p*-value for pairwise comparisons			
Plate *vs.* nail	*p* <0.001		
Plate *vs.* hybrid	*p* <0.001		
Nail *vs.* hybrid	*p* = 0.014		

### Compound cyclic load until failure test

**FIGURE 4:**
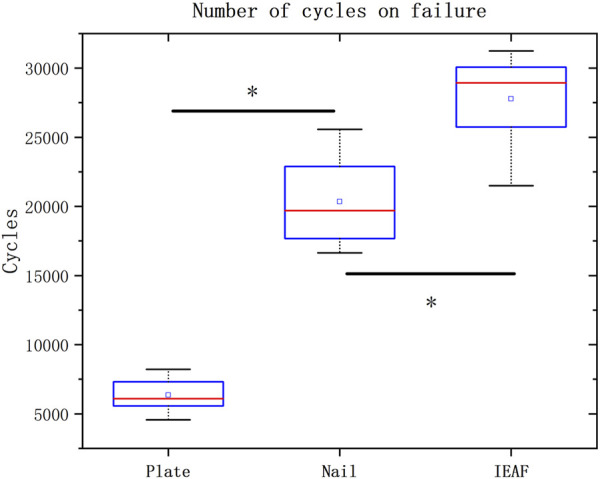
Box and whisker plots showing the values of the number of cycles loaded to failure.

All samples completed 5,000 cycles of loading, except for one sample from the locking plate group. All specimens in the locking plate group experienced internal fixation failure before completing 10,000 cycles. Failure mode in all specimens was the closure of the fracture gap. The number of cycles loaded to failure is presented as the median and the range, and as the mean and the standard deviation in Table 3. Box and whisker plots are depicted with *p*-values when the differences are significant in Figure 4. The IEAF group (27776.75 ± 3229.941) demonstrated the greatest cycles, a significant difference from the locking plate (6344 ± 1126.015; *p* <0.001), and the intramedullary nail (20339.38 ± 3005.853; *p* = 0.002). There was a significant difference between the locking plate and the intramedullary nail (*p* <0.001).

**TABLE 3:** Number of cycles on the failure and displacements of predetermined measurement points for all three constructs in the compound cyclic load test.

	Plate	Nail	Hybrid
Number of cycles on failure			
Mean	6,344 ± 1,126.015	20,339.38 ± 3,005.853	27,776.75 ± 3,229.941
Median	6,098 (4,570–8,215)	19,694 (16,645–25,566)	28,929.5 (21,500–31,240)
*p*-value for the multiple-group comparison		*p* <0.001	
*p*-value for pairwise comparisons			
Plate *vs.* nail	*p* <0.001		
Plate *vs.* hybrid	*p* <0.001		
Nail *vs.* hybrid	*p* = 0.002		
Displacements of predetermined measurement points at 5,000 cycles			
Mean	8.56 ± 0.855	2.986 ± 0.656	2.167 ± 0.541
Median	8.63 (7.2–10)	2.83 (2.31–4.4)	2.127 (1.499–3.191)
*p*-value for the multiple-group comparison		*p* <0.001	
*p*-value for pairwise comparisons			
Plate *vs.* nail	*p* <0.001		
Plate *vs.* hybrid	*p* <0.001		
Nail *vs.* hybrid	*p* = 0.07		
Displacements of predetermined measurement points at 10,000 cycles			
Mean		4.044 ± 1.104	2.734 ± 0.448
Median		4.262 (2.626–6.164)	2.667 (2.004–4.428)
Nail *vs.* hybrid		*p* = 0.027	

**FIGURE 5:**
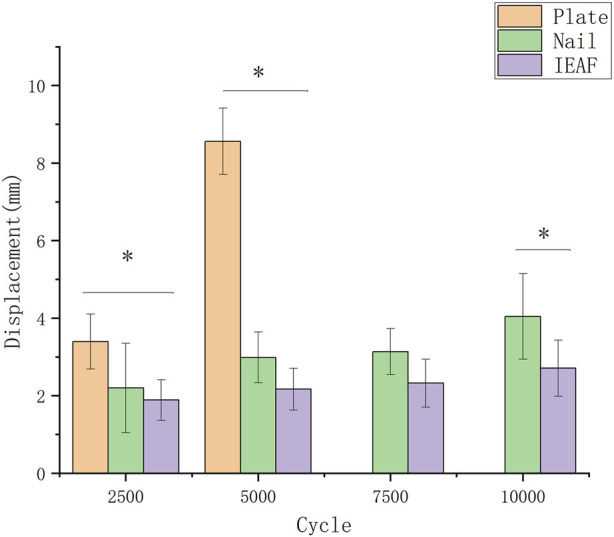
Bar graph showing the values of the relative displacement (in millimeters) of predetermined measurement points. The bars indicate the mean, and the error bars indicate the standard deviation.

The relative displacements of the predetermined pair of measurement points for the three groups are presented as the median and the range, and as the mean and the standard deviation in Table 3. Bar graphs are depicted with *p*-values when the differences are significant in Figure 5. The mean relative displacement value of measurement points in the IEAF group was smaller than that in the other two groups. However, there was no significant difference between the IEAF and the intramedullary nail in the first 7,500 cycles. The mean relative displacement of the intramedullary nail group (3.136 mm) exceeded 3 mm at 7,500 cycles of loading, while the IEAF group (2.709 mm) did not.

## Discussion

In this study, a three-part fracture model of the proximal humerus with osteoporosis was established, and the biomechanical properties of the intra- and extramedullary assembly fixation system, LLPs, and IMNs were compared. Our data showed that the new IEAF system would be stiffer and more durable than the others. This is because the intramedullary part of the intra- and extramedullary assembly fixation system reconstructs the integrity of the medial column, which may distribute much more stress on the LLP. A shorter lever arm can favorably counteract the force on the humeral head from the shoulder glenoid and reduce the risk of the varus displacement of the humeral head compared to the LLP alone. Furthermore, compared to IMNs, a larger contact area between the implant and the bone enables the form of a stronger purchase of the implant in the humeral head to counteract the varus displacement force.

The reconstruction of the medial column integrity improves the lateral locking plate fixation’s mechanical stability in treating elderly PHFs with posteromedial comminution. Due to osteoporosis, severely displaced and comminuted PHFs with bone impaction are common injuries. It is difficult to obtain stable fixation with LLPs in the treatment of elderly PHFs with posteromedial comminution because of the non-axial fixation of LLPs and the osteoporotic bone in elderly people (Zhao et al., 2019). To achieve medial stability, Wanner et al. (2003) proposed a technique with one-third of tubular plates positioned ventrally. Wang et al. (2021) suggested the combination of the medial anatomical locking plate and the lateral plate for the treatment of PHFs. However, the aforementioned methods pose the risk of vascular injury and medial incision infections. Endosteal fibula augmentation with LLPs has been a promising method for reconstructing the medial column and effectively improving the fixation strength of LLPs (Gardner et al., 2008; Little et al., 2014; Kim et al., 2018; Dasari et al., 2022). However, there are problems, such as the high variability of support sites and limited sources, in the application of endosteal fibular augmentation (Little et al., 2014; Wang et al., 2019). Therefore, an intra- and extramedullary assembly fixation was introduced, achieving good clinical results in our patients (Bai et al., 2022; Chen et al., 2023). In this study, we found that initial stiffness (245.12%) and cyclic loading cycles (337.84%) were increased, and gap deletion (74.68%; 5,000 cycles) was reduced in the IEAF group compared to the lateral locking plate group. These data indicate that IEAF would be more durable and stiffer than the lateral locking plate alone for the treatment of PHFs with medial comminution. First, the intramedullary support nail reconstructs the medial column and shares much more force with the plate, which is transferred through the extramedullary to the intramedullary path. This stress transmission trajectory makes the fixation construct much more reasonable. Second, the intramedullary support nail with a shorter lever arm can directly resist the pressure exerted on the humeral head from the scapular glenoid, thus avoiding the varus of the humeral head and reducing postoperative complications.

Strong purchase of the implants in the bone of the humeral head improves intramedullary nail fixation’s mechanical stability in treating elderly PHFs with posteromedial comminution. As an axial fixation method, IMNs showed better mechanical stability in transmitting the vertical force of the medial humerus and maintaining the neck-shaft angle. However, the complication rate of the retraction of the intramedullary nail from the entry point and the loss of reduction of the humeral head was high in the elderly (Plath et al., 2019). These results may be attributed to the weak holding power of screws, especially in the osteoporotic humeral head. The bone quality is usually poor, and it is difficult to carry out secure fixation. Increasing the number of additional screw-in-screws and using cement augmentation may be a method to enhance biomechanical stability (Rothstock et al., 2012; Grünewald et al., 2019). However, screw purchase, which allows good fixation in normal bone, is limited in osteoporotic bone (Kuhn et al., 2014). Chen, in his biomechanical study, found that the intramedullary anatomical support strut provides excellent biomechanical stability (Chen et al., 2020). Stefano showed that intramedullary nail and lateral locking plate assembly fixation exhibit a particular biomechanical advantage in the cases of osteoporotic bones (Brianza et al., 2010). Based on these, the IEAF device appears to have beneficial characteristics that could reduce those severe complications for patients. One possible reason is that the intramedullary part is shaped according to the medullary cavity’s geometry; its increased surface area might significantly improve the support effectiveness for the humeral head. The second reason is that the method of implantation of the intramedullary part is different from that of the traditional intramedullary nail. It does not pass through the articular surface of the humerus apex but through the lateral fracture window, thus avoiding the retraction of the nail from the entry point. The last one is that the role of the proximal anchor point of IMNs potentially counteracting varus displacing forces is replaced by the lateral locking plate, thus reducing the risk of a rotator cuff tear. The data in this study have confirmed the efficacy of the IEAF; we found that initial stiffness (28.8%) and cyclic loading cycles (36.57%) were increased, and gap deletion (32.39%; 10,000 cycles) was reduced in the intra- and extramedullary assembly fixation group compared to the IMN group. The mean relative displacement of the intramedullary nail group (3.136 mm) exceeded 3 mm at 7,500 cycles of loading; at this time point, the intramedullary nail was already engaging the virtual scapular-humeral joint, causing impingement of the supraspinatus tendon.

There are some limitations in this study, which are common in most studies on biomechanical cadaver specimens. Due to the inherent biological variability of the cadaveric specimen, the results for cadaveric specimens had large variations. We approached this problem by reducing the differences between the three groups of specimens in BMD and the humeral head diameter until there was no longer a discernible difference between them. Second, shoulder motions in humans are quite intricate. It is exceedingly difficult to completely recreate the forces operating on the humerus *in vivo* since they are not likely to be unidirectional. Therefore, we applied partial supraspinatus tendon tension while simulating humeral pressure at 25° of abduction to make the results clearer and more clinically applicable. Third, this study cannot fully explain the mechanical conduction and force changes of different configurations of fixation methods, and a finite element analysis is required in the later stage. Lastly, this study did not compare the three biomechanical models against a healthy humeral bone group.

## Conclusion

Under the stress condition of applying partial supraspinatus tendon tension and simulating 25° of humerus abduction, axial fixation structures can provide better mechanical stability than non-axial fixation structures. The intra- and extramedullary assembly fixation is better able to prevent the varus collapse and subsequent screw cut-out.

## Data Availability

The original contributions presented in the study are included in the article/Supplementary Material; further inquiries can be directed to the corresponding authors.
